# The effects of macro‐algae supplementation on serum lipid, glycaemic control and anthropometric indices: A systematic review and meta‐analysis of clinical trials

**DOI:** 10.1002/edm2.439

**Published:** 2023-07-19

**Authors:** Shahla Rezaei, Saeid Doaei, Reza Tabrizi, Saeed Ghobadi, Morteza Zare, Maryam Gholamalizadeh, Zohreh Mazloom

**Affiliations:** ^1^ Student Research Committee Shiraz University of Medical Sciences Shiraz Iran; ^2^ Nutrition Research Center, School of Nutrition and Food Sciences Shiraz University of Medical Sciences Shiraz Iran; ^3^ Department of Community Nutrition, Faculty of Nutrition and Food Technology, National Nutrition and Food Technology Research Institute Shahid Beheshti University of Medical Sciences Tehran Iran; ^4^ Non‐Communicable Diseases Research Center Fasa University of Medical Sciences Fasa Iran; ^5^ Institute for Physical Activity and Nutrition, School of Exercise and Nutrition Sciences Deakin University Melbourne Victoria Australia; ^6^ Cancer Research Center Shahid Beheshti University of Medical Sciences Tehran Iran; ^7^ Department of Clinical Nutrition, School of Nutrition and Food Sciences Shiraz University of Medical Sciences Shiraz Iran

**Keywords:** glycaemic control, integrative medicine, lipid profile, macro‐algae, meta‐analysis

## Abstract

**Introduction:**

Macro‐algae products have been shown to ameliorate the metabolic disorders state. Thus, highlighting their function as supplementary therapeutic agents can be a novel strategy for clinical therapies. This systematic review and meta‐analysis of clinical trials aimed to summarize the effect of macro‐algae consumption on serum lipid profile, glycaemic control and anthropometric factors.

**Methods:**

In this systematic review and meta‐analysis, a comprehensive search was performed for relevant studies published up to May 2023. The Cochran's *Q* test and *I*‐square (*I* ^2^) tests were used to evaluate heterogeneity across the included studies. The meta‐analysis was conducted using random‐effects model (DerSimonian and Laird), and weighted mean difference (WMD) was considered as the pooled effect size.

**Results:**

Out of 8602 papers in the initial screening, eight clinical trials with a total of 438 participants were included into this meta‐analysis. The results indicated that macro‐algae supplementation significantly decreased serum levels of total cholesterol (TC) (WMD = −6.7 mg/dL; 95% CI: −12.59, −0.80; item = 0.026) and low‐density lipoprotein cholesterol (LDL‐c) (WMD = −8.25 mg/dL; 95% CI: −15.38, −1.12; *p*‐value = .023). There was an increase in level of high‐density lipoprotein cholesterol (HDL‐c) (WMD = 0.48 mg/dL; 95% CI: −2.05, 3.01; *p*‐value = .71) which was not statistically significant. Macro‐algae supplementation reduced body mass index (BMI) (WMD = −0.28 kg/m^2^; 95% CI: −0.96, 0.41; *p*‐value = .426), weight (WMD = −0.39 kg; 95% CI: −3.6, 2.83; *p*‐value = .81), waist circumference (WC) (WMD = −0.52 cm; 95% CI: −2.71, 1.66; *p*‐value = .64), fasting blood sugar (FBS) (WMD = −1.95 mg/dL; 95% CI: −5.19, 1.28; *p*‐value = .24) and HbA1c (WMD = −0.02%; 95% CI: −0.14, 0.09; *p*‐value = .66) in intervention group.

**Conclusions:**

This meta‐analysis indicated that macro‐algae supplementation significantly decreased TC and LDL‐c level. It can also increase HDL‐c level and reduce anthropometric indices and glycaemic control factors.

## Graphical abstract

This meta‐analysis indicated that macro‐algae supplementation significantly decreased total cholesterol, low‐density lipoprotein cholesterol and fasting blood glucose levels, but had no significant effect on triglyceride, high‐density lipoprotein cholesterol and body mass index levels.

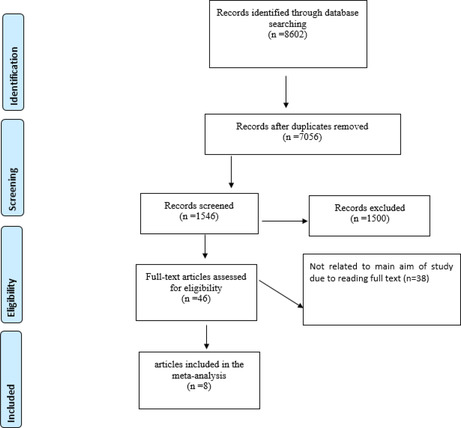

## 1 INTRODUCTION

Prevalent chronic diseases such as diabetes mellitus (DM), cardiovascular disorders and obesity are associated with higher mortality rate worldwide.^1^, ^2^ For instance, type 2 diabetes has become one of the most important metabolic diseases with a prevalence rate of 9%.^3^, ^4^ The common complications linked to the these diseases are hyperglycaemia, insulin resistance, inflammation, heart failure and stroke.^5^ Many factors such as lifestyle and dietary components may influence on diabetes.^6^, ^7^ For example, regular consumption of fruits and vegetables as rich sources of natural antioxidants were reported to decline the risk of DM. In addition, antioxidant compounds like polyphenols have hypoglycaemic effects through the inhibition of carbohydrate digestive enzymes and improving the sensitivity of tissues to insulin hormone.^8^, ^9^, ^10^ Finding alternative functional supplements such as antioxidant compounds to improve the quality of clinical therapies may have beneficial effects against the side effects of chemical drugs.

Recent studies indicated that seaweed polyphenols may have higher biological and pharmacological properties in comparison with other plant‐derived phenolic compounds.^10^, ^11^, ^12^, ^13^ Algae polyphenol‐rich extracts (APREs) were reported to improve the blood glucose and serum lipids levels in a concentration‐dependent manner.^14^, ^15^, ^16^ Interestingly, algal products had no significant side effects^17^ and may be considered as a safe supplementary ingredient for clinical recommendations. Similarly, non‐toxic plant polyphenols are supposed to have a wide range of antidiabetic and anti‐obesity biological properties.^9^, ^18^, ^19^

Recently, marine milieu received more attention as a new sources of active components for enhancing the quality of diet against chronic diseases.^20^ Some edible macroalgae such as *Ascophyllum nodosum*, *Ecklonia cava*, *Ascophyllum nodosum* and *Fucus vesiculosus* have been reported to contain large amounts of polyphenols in their structures.^21^, ^22^, ^23^ Clinical studies also indicated that the consumption of edible seaweed may be effective against metabolic syndrome by modulating a variety of molecular signalling pathways.^14^, ^17^, ^24^ However, the effectiveness of these marine products have not been comprehensively evaluated. So, this systematic review and meta‐analysis aimed to assess the effect of macro‐algae and marine polyphenols (MPs) on serum lipid levels, glycaemic profile and body mass index (BMI).

## 2 MATERIALS AND METHODS

### 2.1 Literature search strategy

PRISMA guidelines (Preferred Reporting Items for Systematic Reviews and Meta‐Analyses) were applied as a framework for reporting this meta‐analysis of Randomized Clinical Trials (RCTs).^25^ The authors systematically searched the online databases including Scopus, Embase, PubMed, ProQuest and ISI for relevant clinical trials that investigated the effects of macro‐algae supplementation on human lipid profiles, fasting blood glucose (FBG), HbA1c and anthropometric factors from the inception up to May 2023. The searches were conducted using the following Mesh keywords:

(‘Seaweed’ OR ‘macro‐algae’ OR ‘marine algae’) AND (‘Clinical Trials as Topic’ OR ‘intervention’ OR ‘intervention*’ OR ‘trial’ OR ‘randomized’ OR ‘placebo’ OR ‘random’ OR ‘randomly’ OR ‘assignment’ OR ‘clinical trial’ OR ‘parallel’ OR ‘cross‐over’ OR ‘RCT’). Additionally, a manual search of reference lists was performed to collect the studies that were not included by the online search.

### 2.2 Inclusion and exclusion criteria

After removing all duplicates reports, two authors (SH. R and S. GH) carefully screened all collected papers according the titles and abstracts to find eligible studies. Next, all eligible studies were categorized based on their findings and contents. Authors (S. GH and M. Z) carefully checked all papers independently and only studies which met the following criteria were included in this meta‐analysis: (i) articles in English language, (ii) papers which included population higher than 18 years old, (iii) RCTs, (iv) papers with enough data (i.e., mean SD [standard deviation], SE [standard error] and CI [confidence interval]) parameters in the beginning and the end of study for both intervention and control participants to measure the targeted factors including glucose and lipid profiles, and (v) intervention duration was equal or higher than 1 week. The outcomes were included in the final analysis if the number of documents related to the outcome was equal or more than three documents. Studies were also excluded if they investigated animal models, published as conference abstract, book chapter, editorials, and patents, or reported insufficient data about the outcome.

### 2.3 Data extraction

The following data of selected articles were separately extracted by two authors (SH. R and S. GH), using a standard form included: the name of the first author, study place, the number of participants involved in both the intervention and control groups, the type and dose of supplementation with alga (g/day or mg/day), algal polyphenol concentration (mg/day), duration of intervention, the age and sex of the participants, type and design of the study, the outcomes of algal products supplementation and the related data for meta‐analysis.

### 2.4 Quality assessment

Sh. R and Z. M assessed the quality of eligible included studies using Cochrane collaboration risk of bias tool.^17^ Discrepancies between the researchers at each stage of quality assessment were resolved based on consensus or discussion with a third author (R. T).

### 2.5 Statistical analysis

Stata version 13.0 (Stata Corp LP) was applied to perform statistical analysis. Random‐effects model was employed with a 95% confidence interval (CI) for the calculation of the pooled weighted mean difference (WMD). The value of mean and SD was recorded for each result in the beginning and the end of the study using the calculation of the difference between the values before/after the intervention. The mean differences for the unreported SDs were calculated by applying the following formula:

$$\mathrm{SD}=\sqrt{\left[{\left(\text{SDbaseline}\right)}^{2}+{\left(\text{SDendstudy}\right)}^{2}-\left(2r\times \text{SDbaseline}\times \text{SDendstudy}\right)\right]}$$

In addition, *r* was calculated and estimated between 0 and 1 values.^26^ Besides, the formula = $\mathrm{SE}\times \sqrt{n}$, where *n* = the number of individuals in each group, was used to measure SD parameter in those cases that reported SE instead of SD. The statistical heterogeneity between trials was calculated using *p* < .05 and *I* ^2^ > 50%. The sensitivity analysis was performed to evaluate the effects of each study on the pooled effect size by using ‘one‐study‐removed’ method (Appendices S1 and S2). Egger's test and funnel plot methods were used to evaluate the publication bias. However, subgroup analysis was not performed for some parameters due to insufficient number of studies.

#### 2.5.1 Certainty of evidence assessment

The overall assessment of the evidence for outcomes was conducted using the modified Grading of Recommendations Assessment, Development and Evaluation (GRADE) approach. This evaluation was performed by two independent investigators (Sh. R and R. T). Using this approach, the certainty of the body of evidence is assessed against factors such as the presence of risk of bias, inconsistency, indirectness, imprecision and evidence of publication bias.

## 3 RESULTS

**FIGURE 1:**
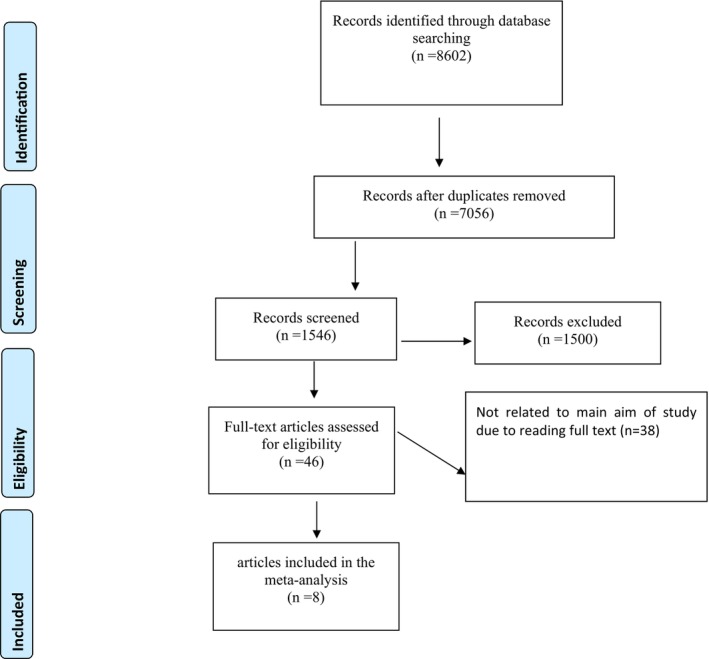
Flow chart of the included studies, including identification, screening, eligibility and the final sample included.

The process of the identification of the related studies is presented in Figure 1. A total of 8602 citations were initially screened. Finally, after title and abstract screening, removing duplication citations, and checking the full texts of related studies, eight eligible clinical trials^10^, ^14^, ^20^, ^27^, ^28^, ^29^, ^30^, ^31^ published between 2008 and 2023 were included in this meta‐analysis. No extra paper was added by hand searching or from the other sources.

**FIGURE 2:**
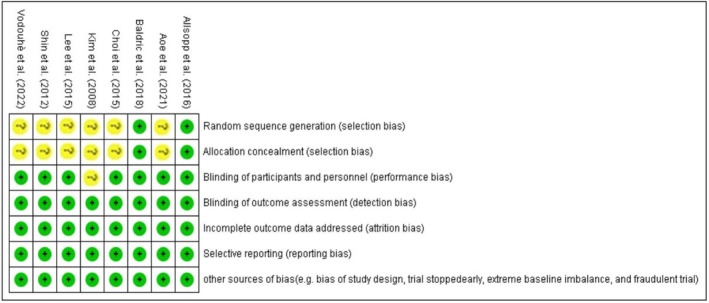
The methodological quality of the included eligible studies (for each risk of bias item).

Overall, 438 participants were included within this systematic review. The age of the participants was between 18 and 80 years old, and both of men and women were considered. One study by Shin et al.^14^ investigated the effect of two different concentrations of algae on two separated groups, but only the dose of high dose group was similar to the dose used in other studies included in the meta‐analysis and only this group of algal supplementation was considered in the final analysis. Also, no significant toxicity or side effect was reported for the consumption of algal products in the included studies for meta‐analysis. All PICO (Patient/Problem, Intervention, Comparison and Outcome) components are presented in Table 1. The assessment of the method quality of included studies by the author's judgement for each risk of bias items using the Cochrane collaboration risk of bias tool is shown in Figure 2.

**TABLE 1:** Characteristics of the studies included in the systematic review.

Author, year	Country	Intervention/Control (*N*)	Type of intervention	Type of seaweed	PPs dose (mg)	Duration (weeks)	Clinical condition	Subjects age (years)/Sex	Study design	Significant out comes
Kim et al.,^29^ 2008	Korea	10/10	48 g seaweed supplementation 3 times/day	Powdered sea tangle and sea mustard as seaweed	NR	4	Type 2 diabetes mellitus & BMI < 35 & FPG > 150 mg/dL	40–70/M & F	Randomized, controlled clinical trial	A significant reduction of FBG, 2‐hppG, TG and increases of HDL‐C in seaweed group. TC and LDL‐C were not affected by seaweed.
Shin et al.,^14^ 2012	Korea	32/32	144 mg ECP/day supplementation with a polyphenol extract from ECP	ECP (brown alga)	144	12	Subjects with BMI = 24–29	19–55/M & F	Randomized, double‐blind, placebo‐controlled trial with parallel‐group design	A significant decreases in BMI, body fat ratio, WC, waist/hip ratio, TC, LDL‐C, TC/HDL‐C, AI, serum glucose and systolic blood pressure, and a significant increase in serum HDL‐C in HD group as compared with the placebo group.
Choi et al.,^28^ 2015	Korea	33/30	400 mg/day extract of *Ecklonia cava* (ECE)	ECP (brown alga)	NR	12	Healthy subjects with TC > 200 mg or LDL‐C > 110 mg	19–80/M & F	Randomized, double‐blind and placebo‐controlled trial	The serum TC, LDL‐C, HDL‐C, TG and waist‐hip ratio levels were significantly lowered in the ECE group as compared to the placebo.
Lee et al.,^30^ 2015	Korea	36/37	1500 mg/day of AG‐dieckol	Dieckol‐rich extract from brown algae, ECP	690	12	Prediabetic adults with FPG = 100–180 mg	20–65/M & F	Randomized, double‐blind, placebo‐controlled trial with parallel‐group design	A significant decrease in 2‐hppG, insulin and C‐peptide level.
Allsopp et al.,^20^ 2016	Ireland	16/20	5 g/day *P. palmata* incorporated into a bread	*Palmaria palmata*	NR	4	Healthy participants	18–65/M & F	Double‐blind, randomized placebo‐controlled human intervention	A significant increase in serum CRP and TG in *P. palmata* group.
Baldrick et al.,^27^ 2018	United Kingdom	78/78	400 mg/day capsule containing 100 mg seaweed polyphenol and 300 mg/day maltodextrin	Brown seaweed (*Ascophyllum nodosum*)	100	8	Participants with BMI ≥25	30–65/M & F	Randomized, double‐blind, placebo‐controlled crossover trial	Decrease in DNA damage. No significant changes in CRP, antioxidant status, or inflammatory cytokines.
Aoe et al.,^31^ 2021	Japan	24//24	Kelp powder (Laminaria), 10 tablets orally, 3 times/day	Seaweed, kelp powder (Laminaria)	NR	8	Subjects with BMI = 25–30 kg/m^2^	40–65/M & F	Randomized, double‐blind, placebo‐controlled intervention study	Body fat percentage was significantly decreased in male subjects from the test group compared with the placebo group. The same tendency was observed for body weight and BMI in male subjects.
Vodouhè et al.,^10^ 2022	Canada	27/29	500 mg/day of brown seaweed extract	Brown seaweed extract rich in Polyphenols (*Ascophyllum nodosum* and *Fucus vesiculosus* extract)	175	12	Overweight and obese prediabetic subjects	18–70/M & F	Randomized, placebo‐controlled, double‐blind, and parallel clinical trial	Consumption of brown seaweed extract had no effect on body weight or blood glucose. An early attenuation of the inflammatory response was observed in association with marginal changes in metabolic parameters related to the prevention of diabetes type 2.

Abbreviations: 2‐hppG, 2‐h postprandial glucose; AI, atherogenic index; BMI, body mass index; CRP, c‐reactive protein; DBP, diastolic blood pressure; DNA, Deoxyribonucleic acid; ECE, *Ecklonia cava* extract; ECP, *E. cava* phenol; F, female; FBG, fasting blood glucose; HD, high dose; HDL, high‐density lipoprotein; LD, low dose; LDL, low‐density lipoprotein; M, male; *N*, number; NR, no reported; PPs, polyphenols; SBP, systolic blood pressure; TC, total cholesterol; TG, triglyceride; WC, waist circumference.

### 3.1 The effect of macro‐algae on the outcomes

By consumption of macro‐algae, serum levels of TC (WMD = −6.7 mg/dL; 95% CI: −12.59, −0.80; *p*‐value = .026; *I* ^2^ = 31.03%, *p*‐value = .29) and low‐density LDL‐c (WMD = −8.25 mg/dL; 95% CI: −15.38, −1.12; *p*‐value = .023; *I* ^2^ = 50.45%, *p*‐value = .046) were significantly decreased and serum level of HDL‐c increased (WMD = 0.48 mg/dL; 95% CI: −2.05, 3.01; *p*‐value = .71; *I* ^2^ = 68.14%, *p*‐value = .042) in the intervention group compared with the placebo group which was not statistically significant. There was no significant change in TG level (WMD = 6.44 mg/dL; 95% CI: −6.56, 19.45; *p*‐value = .33; *I* ^2^ = 0.00%, *p*‐value = .44).

In terms of glycaemic control, macro‐algae consumption decreased serum levels of FBS (WMD = −1.95 mg/dL; 95% CI: −5.19, 1.28; *p*‐value = .24; *I* ^2^ = 41.41%, *p*‐value = .12) and HbA1c (WMD = −0.02%; 95% CI: −0.14, 0.09; *p*‐value = .66; *I* ^2^ = 0.00%, *p*‐value = .46) in the intervention group compared to placebo group.

**FIGURE 3:**
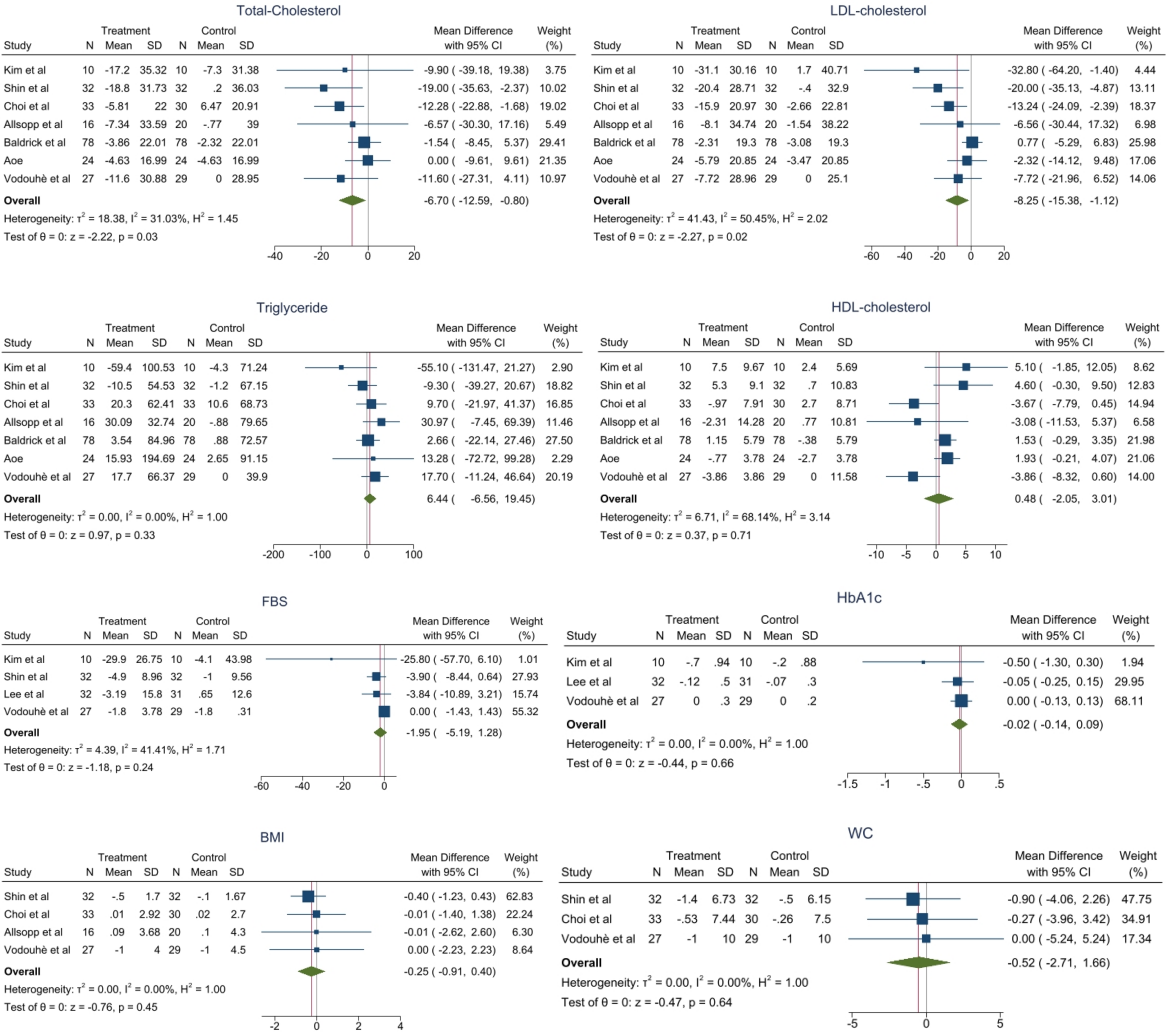
Forest plot assessing the effect of seaweed supplementation on studied outcomes (the *p*‐values are related to heterogeneity not outcomes).

BMI level (WMD = −0.25 kg/m^2^; 95% CI: −0.91, 0.40; *p*‐value = .45; *I* ^2^ = 0.00%, *p*‐value = .96), weight (WMD = −0.39 kg; 95% CI: −3.6, 2.83; *p*‐value = .81; *I* ^2^ = 0.00%, *p*‐value = .96) and waist circumference (WC) (WMD = −0.52 cm; 95% CI: −2.71, 1.66; *p*‐value = .64; *I* ^2^ = 0.00%, *p*‐value = .95) were decreased in intervention group, but it was not statistically significant. The forest plots for the effect of macro‐algae supplementation on studied outcomes are shown in Figure 3.

### 3.2 Publication bias and sensitivity analysis

**FIGURE 4:**
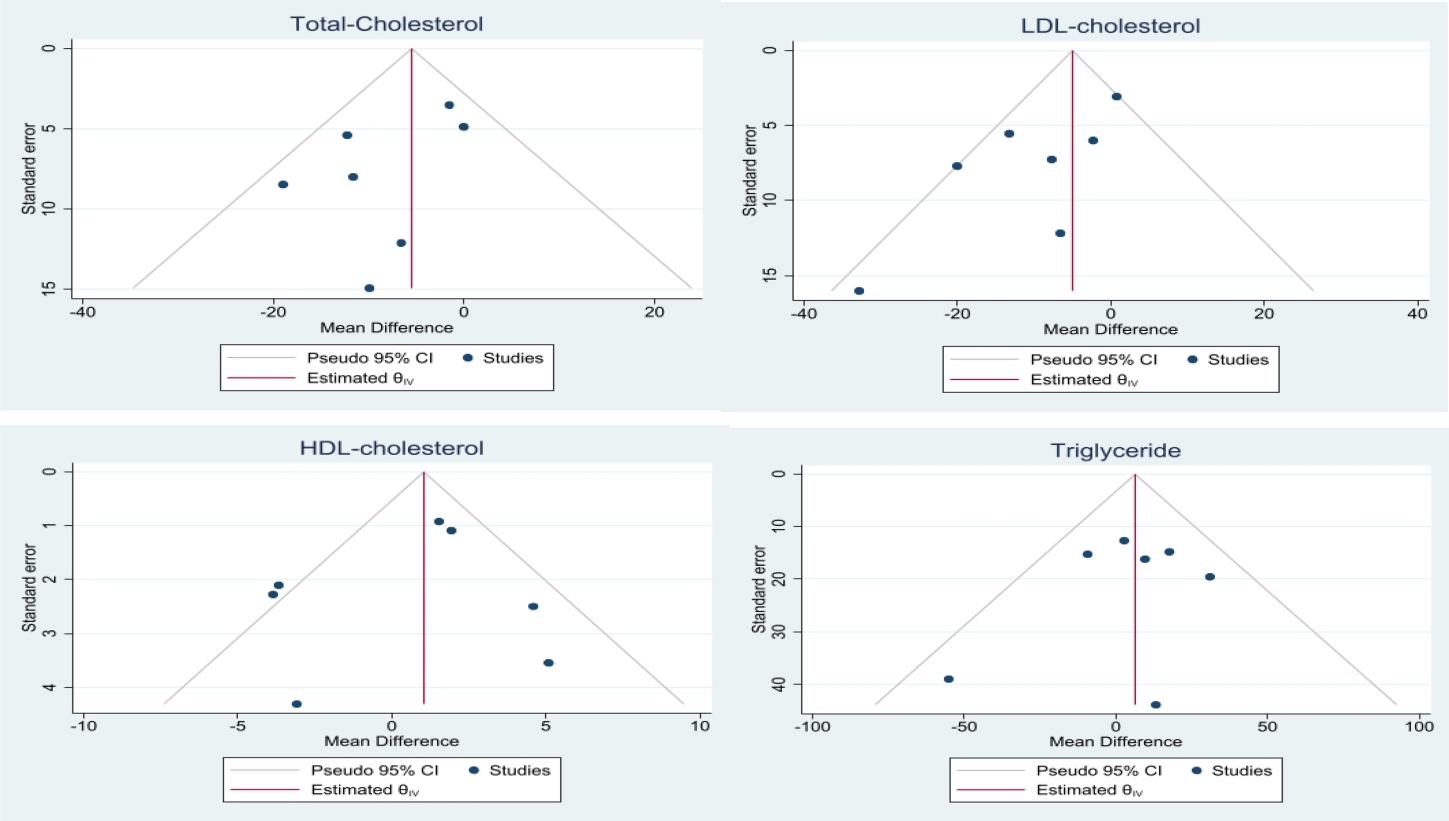
Funnel plot assessing the publication bias.

Findings from sensitivity analysis did not show considerable effects of the macro‐algae on levels of lipid profiles, glycaemic control and anthropometric indices after excluding each study from the pooled effect sizes. There was no evidence of publication bias (Figure 4) among the included articles assessing the effect of macro‐algae on TC (*p* Egger's = .18), LDL‐c (*p* Egger's = .057), HDL‐c (*p* Egger's = .8) and TG (*p* Egger's = .59).

#### 3.2.1 Certainty of evidence

Table 2 shows the certainty of body evidence regarding outcomes using a modified GRADE approach. The certainty of the evidence was evaluated as moderate in two outcomes, low and very low for three considered outcomes. The presence of the risk of bias, indirectness and imprecision were the main factors downgrading the certainty of the evidence.

**TABLE 2:** Summary of findings using modified GRADE profile.

Pooled effect MD (95% CI)	No. of studies	Study design	Risk of bias	Inconsistency	Indirectness	Imprecision	Publication bias	Certainty of evidence
Studied outcomes								
Total cholesterol								
−6.70 (−12.59, −0.80)	7	Clinical trial	0	0	−1^a^	0	0	+ + + − (Moderate)
LDL cholesterol								
−8.25 (−13.38, −1.12)	7	Clinical trial	0	−1	−1	0	−1	+ − − − (Very low)
Triglyceride								
6.44 (6.56, 19.45)	7	Clinical trial	0	0	−1	0	0	+ + + − (Moderate)
HDL cholesterol								
0.48 (−2.05, 3.01)	7	Clinical trial	0	−1^b^	−1	0	0	+ + − − (Low)
FBS								
−1.95 (−5.19, 1.28)	4	Clinical trial	0	0	−1	−1	0	+ + − − (Low)
HbA1c								
−0.02 (−0.14, 0.09)	3	Clinical trial	0	0	−1	−2^c^	0	+ − − − (Very low)
BMI								
−0.25 (−0.91, 0.40)	4	Clinical trial	0	0	−1	−1	0	+ + − − (Low)
WC								
−0.52 (−2.71, 1.66)	3	Clinical trial	0	0	−1	−2	0	+ − − − (Very low)

*Note*: The symbols + + − − show the certainty of evidence.

Abbreviations: BMI, body mass index; CI, confidence interval; FBS, fasting blood glucose; GRADE, grades of recommendation, assessment, development, and evaluation; MD, mean difference; WC, waist circumference.

^a^ Down‐graded one level as the indirectness was considerable.

^b^ Down‐graded one level as the inconsistency (*I* ^2^) was more than 50%.

^c^ Down‐graded one level as the imprecision was considerable.

## 4 DISCUSSION

The results of this meta‐analysis of RCTs indicated that macro‐algae supplementation significantly reduced TC and LDL‐C concentrations levels. Macro‐algae supplementation also had a reducing effect on anthropometric indices and glycaemic control factors, and an increasing effect on HDL‐C level but were not statistically significant. Studies suggested that macro‐algae have particular classes of polyphenols called phlorotannins (e.g., eckols, fuhalols, fucophlorethols, phlorethols, fucols and isofuhalos)^11^, ^32^ which act as free radicals and reactive oxygen species (ROS) scavenger and can influence various types of chronic human diseases such as diabetes mellitus, Alzheimer's disease, cancers through inhibition or activation of specific targets or pathways. Some studies reported that individuals who consumed diets enriched with considerable amounts of seaweeds products have higher chance to survive from chronic diseases.^33^ Macro‐algae polyphenols could modulate the activity of many key enzymes involved in human chronic disorders like diabetes in a dose‐dependent manner.^24^

Zhao et al.^34^ reported that algal bioactive metabolites showed hypoglycaemic benefits through inhibition of carbohydrate digestive enzymes (either α‐amylase or α‐glucosidase), the inhibition of postprandial hyperglycaemia, the activation of Akt and AMP‐activated protein kinase signalling cascades, the increasing/improving of insulin secretion, the improving insulin sensitivity, delaying glucose absorption and the inhibition of protein tyrosine phosphatase 1B signalling pathway in a dose‐dependent manner. Other studies reported that the oral administration of high fat diet‐induced obese mice (HFD‐induced mice) with 500 mg of *E. cava* polyphenol‐rich extract could regulate lipid metabolism and inflammatory pathways.^35^ Also, this extract decreased body weight gain and induced antioxidant defence systems in studied animals. In another paper, Kojima‐Yuasa^11^ described that the phenolic compounds of *E. cava* are effective agents for decreasing the blood glucose level and preventing hyperglycaemia‐induced oxidative stress. Herein, the outcomes of this meta‐analysis identified that the short‐term consumption of whole product of brown macro‐algae or its polyphenol‐rich extracts could increase the level of HDL‐C and decrease the level of TC, LDL‐C and glycaemic control profiles. In addition, these macro‐algae products also improved anthropometric factors but the result was not statistically significant.

In recent systematic review by Murray et al.,^36^ authors described that algal phenolic compounds could relatively reduce the level of FBG, TC and LDL in patients, which was in line with this study.^36^ From these results, it seems that APREs are effective against blood glucose level and serum lipid profile, and therefore, these functional marine ingredients can be potential sources of supplementary agents for improving the health status of diabetic and obese patients or individuals suffered from cardiovascular disorders. As mentioned previously, inconsistent effects were observed on the effects of APREs on BMI, and this type of supplementation has apparently few or no effects on these parameters.

In addition, short‐term macro‐algae supplementation had no significant effect on triglyceride, HDL‐c and anthropometric factors. No clear mechanism has been reported in the literature, and further long‐term clinical studies will be required to accurately determine the exact effect of these compounds on anthropometric indices. Some studies failed to find the effect of algae on serum glucose and lipid profiles and anthropometric indices. Various factors such as individual characteristics of the subjects, clinical conditions of the participants, duration of the intervention, dose–response effect, and type of seaweed and polyphenols can affect the relationship between algae and health outcomes. For example, Kim et al.^29^ reported that TC and LDL‐c were not affected by seaweed. However, their study was performed on a small sample size of 10 people in the intervention group and 10 people in the control group and also in a short period of time (1 month), which may affect the strength of the results.

With the increasing interest on developing new drugs for diabetes mellitus, searching for natural drug‐like compounds opened new windows in nutritional and pharmacological studies. Studies on macro‐algae polyphenols suggested that these compounds may be beneficial in the prevention of metabolic diseases.

This study had some limitations. The major limitation of this meta‐analysis was the short duration of the included studies. Another important limitation factor is the small number of participants recruited for performing clinical trials. Furthermore, not enough studies have been done on some of the important outcomes yet, and we have not been able to perform subgroup analysis. Further trials with larger populations and longer duration are needed to confirm the beneficial effects of seaweeds against the metabolic diseases.

## 5 CONCLUSION

The outcomes of the current meta‐analysis suggest that the short‐term administration of algal products either through consuming whole products or polyphenol‐rich extracts could decrease the TC and LDL‐c levels. Macro‐algae polyphenol‐rich extracts can be considered as new safe supplementary products for ameliorating complications associated with DM and obesity. However, further investigations in this field should evaluate the large‐scale clinical effect of seaweeds polyphenols to more entirely understand their potential.

## AUTHOR CONTRIBUTIONS

**Shahla Rezaei:** Software (equal). **Reza Tabrizi:** Supervision (equal); validation (equal). **Saeed Ghobadi:** Formal analysis (equal). **Morteza Zare:** Writing – original draft (equal). **Zohreh Mazloom:** Formal analysis (equal); software (equal). **Saeid Doaei:** software (equal), **Maryam gholamalizadeh:** Writing ‐ original draft (equal).

## FUNDING INFORMATION

Funding for this study was provided by Shiraz University of Medical Sciences.

## CONFLICT OF INTEREST STATEMENT

All authors read the manuscript before the submission process, and they declared that there was no conflict of interest to list their name in this paper.

## ETHICS STATEMENT

The study was approved by the ethics committee of Shiraz University of Medical Sciences, Shiraz, Iran.

## Supporting information


Appendix S1

Appendix S2

## Data Availability

All data will be made available upon request.
