# Supplementary material for: The effects of macro‐algae supplementation on serum lipid, glycaemic control and anthropometric indices: A systematic review and meta‐analysis of clinical trials
Source: Endocrinol Diabetes Metab. 2023 Jul 19;6(5):e439. doi: 10.1002/edm2.439 (PMC10495559; doi:10.1002/edm2.439)
Supplement: Supplementary file 1 — Appendix S1 [file EDM2-6-e439-s001.docx]

Till 14/May/2023

**Pubmed= 594**

(((((Seaweed[MeSH Terms]) OR (Seaweed[Title/Abstract])) OR (macro-algae[Title/Abstract])) OR (macroalgae[Title/Abstract])) OR (marine algae[Title/Abstract])) AND ((((((((((((((((Clinical Trials as Topic[MeSH Terms]) OR (Clinical Trials as Topic[Title/Abstract])) OR (intervention[Title/Abstract])) OR (intervention*[Title/Abstract])) OR (trial[Title/Abstract])) OR (randomized[Title/Abstract])) OR (placebo[Title/Abstract])) OR (random*[Title/Abstract])) OR (assignment[Title/Abstract])) OR (clinical trial[Title/Abstract])) OR (Clinical Trial [Publication Type])) OR (parallel[Title/Abstract])) OR (cross‐over[Title/Abstract])) OR (Cross-Over Studies[MeSH Terms])) OR (Crossover[Title/Abstract])) OR (RCT[Title/Abstract]))

**Scopus= 2496**

TITLE-ABS-KEY ( "Seaweed"  OR  "macro-algae"  OR  "macroalgae"  OR  "marine algae" )  AND  TITLE-ABS-KEY ( "Clinical Trials"  OR  "intervention"  OR  " intervention*"  OR  "trial"  OR  "randomized"  OR  "placebo"  OR  "random*"  OR  "assignment"  OR  "clinical trial"  OR  "parallel"  OR  "cross‐over"  OR  "Crossover"  OR  "RCT" )

**ISI=2874**

TS=(Seaweed OR macro-algae OR macroalgae OR marine algae) AND TS=(Clinical Trials OR intervention OR intervention* OR trial OR randomized OR placebo OR random* OR assignment OR clinical trial OR parallel OR cross‐over OR Crossover OR RCT)

**Proquest=2068**

AB,TI("Seaweed"  OR  "macro-algae"  OR  "macroalgae"  OR  "marine algae") AND AB,TI("Clinical Trials"  OR  "intervention"  OR  " intervention*"  OR  "trial"  OR  "randomized"  OR  "placebo"  OR  "random*"  OR  "assignment"  OR  "clinical trial"  OR  "parallel"  OR  "cross‐over"  OR  "Crossover"  OR  "RCT")

**Embase=570**

(‘Seaweed’:ab,ti OR ‘macro-algae’:ab,ti OR ‘macroalgae’:ab,ti OR ‘marine algae’:ab,ti) AND (‘Clinical Trials’:ab,ti OR ‘intervention*’:ab,ti OR ‘trial’:ab,ti OR ‘randomized’:ab,ti OR ‘placebo’:ab,ti OR ‘random*’:ab,ti OR ‘assignment’:ab,ti OR ‘clinical trial’:ab,ti OR ‘parallel’:ab,ti OR ‘cross‐over’:ab,ti OR ‘Crossover’:ab,ti OR ‘RCT’:ab,ti)
