# Supplementary figures and images for: The effects of macro‐algae supplementation on serum lipid, glycaemic control and anthropometric indices: A systematic review and meta‐analysis of clinical trials
Source: Endocrinol Diabetes Metab. 2023 Jul 19;6(5):e439. doi: 10.1002/edm2.439 (PMC10495559; doi:10.1002/edm2.439)

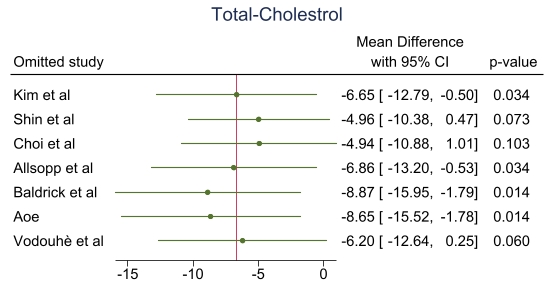

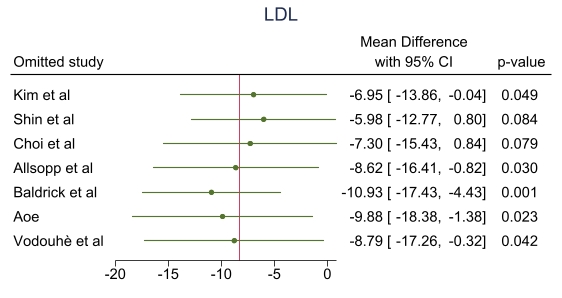

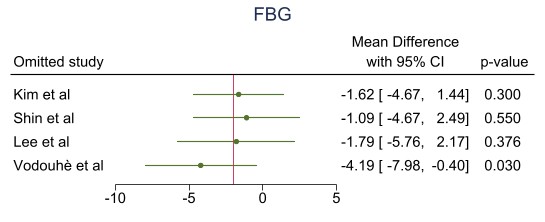

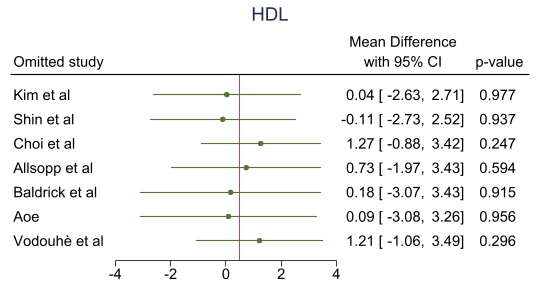

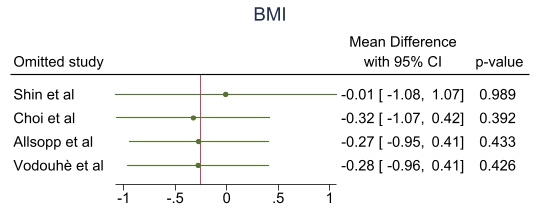

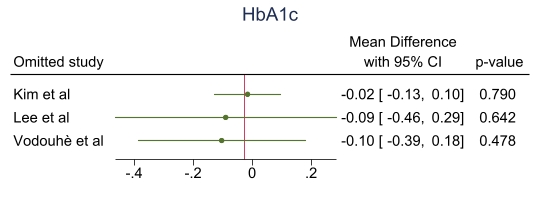

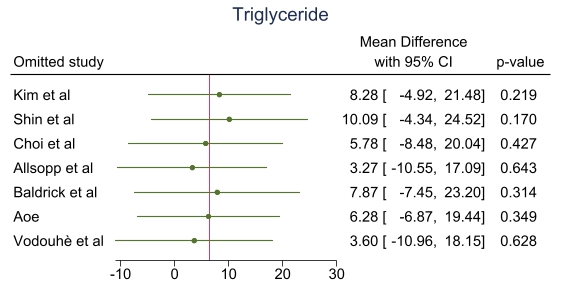

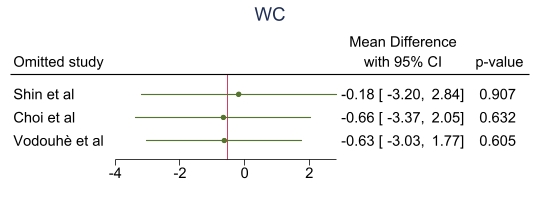

Supplement: Supplementary file 2 — Appendix S2 [file EDM2-6-e439-s002.docx]
